# Behavior-related health risk factors, mental disorders and mortality after 20 years in a working aged general population sample

**DOI:** 10.1038/s41598-023-43669-8

**Published:** 2023-10-05

**Authors:** Ulrich John, Hans-Jürgen Rumpf, Monika Hanke, Christian Meyer

**Affiliations:** 1 Institute of Community Medicine, Department of Social Medicine and Prevention, University Medicine Greifswald, W.-Rathenau-Str. 48, 17475 Greifswald, Germany; 2 German Center for Cardiovascular Research, partner site Greifswald, Greifswald, Germany; 3 Department of Psychiatry and Psychotherapy, Research Group S:TEP, University of Lübeck, Ratzeburger Allee 160, 23538 Lübeck, Germany

**Keywords:** Epidemiology, Risk factors

## Abstract

Mortality is predicted by the sum of behavior-related health risk factors (BRFs: tobacco smoking, alcohol drinking, body overweight, and physical inactivity). We analyzed degrees and combinations of BRFs in their relation to mortality and adjusted for mental disorders. In a random sample of the general population in northern Germany aged 18–64, BRFs and mental disorders had been assessed in 1996–1997 by the Munich Composite International Diagnostic Interview. A sum score including eight ranks of the behavior-related health risk factors was built. Death and its causes were ascertained 2017–2018 using residents’ registration files and death certificates. Relations of the sum score and combinations of the BRFs at baseline with all-cause, cancer, and cardiovascular mortality 20 years later were analyzed. The sum score and combinations predicted all-cause, cardiovascular and cancer mortality. The odds ratio of the sum score was 1.38 (95% confidence interval 1.31–1.46) after adjustment for age, gender, and mental disorder. In addition to the BRFs, mood, anxiety or somatoform disorders were not related to mortality. We concluded that the sum score and combinations of behavior-related health risk factors predicted mortality, even after adjustment for mental disorders.

## Introduction

Data from controlled trials with humans and animal studies revealed that behavior-related health risk factors (BRFs) may have causal effects on health conditions^1^. In a general population sample, lifestyle counseling according to smoking, risky alcohol consumption, diet, and physical inactivity^2^ may have improved mental and physical self-reported health^3^ but did not seem to decrease mortality or ischemic heart disease^4^. Half-yearly advice to stop smoking and diet counselling over a 5-year period in a male general population sample may have added to reduced mortality from myocardial infarction^5^. Animal studies showed effects of diet and exercise interventions on cardiovascular conditions^1,6,7^. Cohort studies are limited to the analysis of potential predictors of mortality. However, different aspects of BRFs may be taken into account such as dose–response relations with mortality risk, concomitants of BRFs and their prevalence.

A dose–response relation between the number of BRFs among tobacco smoking alcohol risk drinking, poor diet, and physical inactivity and mortality exists^8^. A hazard ratio 0.83 (0.80–0.85) for a one-point increment in a healthy lifestyle score of 0 to 4 was found for time to death in a general population sample in the USA (n = 44,462) and almost the same hazard ratio 0.83 (0.82–0.84) in the UK (n = 399,537)^8^. Similar findings come from two cohorts in the USA with a mortality follow-up after more than 20 years. The data of 79,043 women and 39,544 men had been analyzed^9^. With each one-point increment of a healthy lifestyle score, the hazard ratio was 0.81 (0.79–0.83) for all-cause mortality among people who did not use aspirin, blood pressure or lipid lowering medication. According to meta-analysis data, a pooled hazard ratio 0.42 (CI 0.37 to 0.46) was found for cardiovascular^10^, and 0.48 (CI 0.42 to 0.54) for cancer mortality among persons with the most compared to those with the least healthy lifestyle^11^. Adherence to four lifestyle recommendations (non-smoking, low alcohol drinking, staying physically active, and maintaining a healthy diet) was related to a 70% lower hazard of death than following none of these recommendations^12^. In a variety of samples from America, Asia, and Europe the number of BRFs has been found to predict time to death for all-cause^13^, cardiovascular^9,10^, and cancer mortality^11,14^. In a sample of 35 to 70 years old residents across 21 nations including high-, medium- and low-income countries, BRFs have been shown to be related with cardiovascular events and mortality^15^.

Little is known about further factors that might add to the prediction of death in addition to the BRFs from general population samples. Mental disorders belong to these factors. A reason is that a relation between mental disorders and mortality has been shown. A second reason is that BRFs and mental disorders are related to each other. Third, both BRFs and mental disorders are prevalent.

Research revealed that individuals with mental disorders compared to those without have an increased mortality^16^. Among 148 studies reviewed, 135 found higher mortality among subjects with a mental disorder and more years of life lost than among comparison samples^16^. Life expectancy of people with a mental disorder was 10 years lower than in comparison samples^16^. Most of the evidence exists for all-cause and for cardiovascular mortality^17^. But also, a higher mortality was found among psychiatric patients with cancer compared to the general population with cancer^18^.

BRFs and mental disorders are related to each other. BRFs may add to the evolvement and maintenance of a mental disorder or may accrue when maturing out of it. Dietary factors and lack of physical activity have been shown to be associated with depressive^19,20^ and with anxiety disorders^21,22^. Mood and anxiety disorders may add to BRFs in the risk of death^23^. However, there is a lack of general population data.

Both BRFs and mental disorders are prevalent. In the United States of America, in a general population sample 47.3% had two or three among three health risk behaviors (smoking, poor diet, physical inactivity)^24^. Among men at age 18 to 44, there were 17% current smokers, 68% overweight or obese, and 54% did not meet activity recommendations^25^. According to general population data from Great Britain, considerable proportions of the study participants did not meet the criteria for health behavior due to smoking (45.2%), risky alcohol consumption (37.0%), overweight or obesity (66.7%) or physical inactivity (66.7%)^8^. Among an adult general population sample in Germany, 51.5% of the female and 61.9% of the male respondents had two or more of four BRFs (tobacco smoking, risky alcohol consumption, overweight or obesity, physical inactivity)^26^. According to mental disorders, pooled survey data from 85 studies and 39 countries revealed a proportion of 29.2% in general population samples with a mental disorder in lifetime before^27^. Among the population aged 18 to 65 in European countries, 27.1% have been estimated to suffer each year from a mental disorder such as anxiety, depressive, somatoform or substance use disorder^28^. General population data from the United States revealed that 13.2% had a major depressive disorder and 17.2% an anxiety disorder in their lifetime before^29^.

Limitations of the knowledge so far include, first, that hardly anything is known about the prediction of mortality by BRFs and mental disorders in one prediction model with mental disorders being assessed according to international standards. Symptoms of depressive disorders have been analyzed^15,30^. However, the assessment was limited by using a screening instrument^15,30^. Second, a lack of evidence exists concerning associations between BRFs and mental disorders among general population samples. Third, a variety of studies just considered whether single BRFs were present or absent^10^. Three or more ranks of each single BRF and sum scores of these ranks may be expected to provide more information on dose–response relations of BRFs with mortality. Fourth, little evidence exists about specific combinations of single BRFs and mortality^31^.

The aim of the present study was, first, to analyze the mortality risks among a sample of working aged adult residents based on degrees of tobacco smoking, alcohol drinking, body overweight, physical inactivity and prevalent mental disorders that had been assessed 20 years before according the Diagnostic and Statistical Manual of the American Psychiatric Association^32^. Second, more than four ranks among tobacco smoking, alcohol drinking, body overweight, and physical inactivity and the combinations of these BRFs were to be included.

## Methods

### Sample

A random adult population sample aged 18 to 64 years was used^33^. Among the 5,829 individuals eligible for the baseline study, 4,093 (70.2%) interviews had been completed July 1996 to March 1997, and 4,075 were analyzed^33^. A mortality follow-up was conducted from April 2017 until April 2018. Among the 4,075 baseline study participants, vital statistics data could not be proved for 47^34^. For 4,028 (98.8% of 4,075) study participants, we received the data. This is our final sample for the reported data analysis.

### Assessments

#### Baseline

Four BRFs were assessed by self-statements: tobacco smoking, alcohol drinking, body overweight, and leisure time physical inactivity. Tobacco smoking was part of a standardized interview. Alcohol drinking, body overweight and physical inactivity were assessed using standardized questionnaires which were filled in by the study participants as part of the interview. Tobacco smoking included smoking status (never or ever less than daily smoker, former daily smoker, current daily smoker less than 20 cigarettes per day, current daily smoker 20 or more cigarettes per day). Never smokers were those who answered “No” to the question whether they ever had smoked tobacco by cigarette, cigar or pipe in their life before. Ever less than daily smokers reported a history of smoking but not daily over a time period longer than 4 weeks in their life. Former daily smokers were smokers who had disclosed daily tobacco smoking over a time period of more than 4 weeks but not during the last 12 months prior to the baseline interview. Current daily smokers had smoked daily during the last 12 months prior to the interview. Alcohol drinking during the last 12 months prior to the interview was assessed by the Alcohol Use Disorders Identification Test, first three questions (Alcohol Use Disorders Identification Test Consumption, AUDIT-C^35,36^): “1. How often did you have an alcoholic drink in the past 12 months?” (0: never, 1: once a month or less, 2: 2 to 4 times a month, 3: 2 to 3 times a week, 4: 4 times a week or more often). Those who had an alcohol drink in the last 12 months received questions 2 and 3: “2. If you have an alcoholic drink, how many glasses do you typically drink at 1 day?” (a small glass or a bottle of beer, a small glass of wine or sparkling wine, Spirits or liquor; 0: 1–2, 1: 3–4, 2: 5–6, 3: 7–9, 4: 10 or more), “3. How often did you drink 6 or more glasses in a row?” (0: never, 1: less than once a month, 2: once a month, 3: once a week, 4: daily or almost daily). The sum of the three item scores is the AUDIT-C sum score. Body overweight was estimated using the body mass index (BMI) as weight in kilograms divided by height in meters squared. Physical inactivity was assessed by a score with the value range 5 to 20 based on five questions about single areas of leisure time physical activity: ride a bicycle; practice gymnastics, aerobics or dancing; playing sports such as soccer, volleyball, handball, tennis; hiking or going on longer walks; working in house or garden. For each of these activities the respondent was asked to indicate how often s/he practiced the activity. We collapsed the answer categories into four: daily or almost daily, several times per week, once a week, less than once a week or never. We used a score of 1 to 4. It was the higher the less frequently the activity was practiced.

Mental disorders included mood disorders, anxiety disorders, and somatoform disorders^34^. These had been assessed for the lifetime before baseline according to the criteria of the Diagnostic and Statistical Manual of Mental Disorders, fourth edition, American Psychiatric Association^32^. The Composite International Diagnostic Interview (M-CIDI)^33^^,^^37^ had been used.

#### Mortality follow-up

Vital statistics data were retrieved from the residents` registration files at the place of the last residence for all-cause mortality. For cardiovascular and for cancer mortality, we used the death certificate information. Based on the information of the residents’ registration office about the date of death, we received the death certificates from the local health authorities at the place of residence of the individual. The death certificates included health disorders which inferred death, disorders which were a main cause of, and disorders which may have contributed to death. A maximum of 15 disorders and 11 disorders as found by autopsy could be documented by the certificating physician. We used cardiovascular disorders and cancer among the disorders that inferred or were a main cause of death based on the International Classification of the Diseases, version 10^38^.

### Data analysis

The data analysis was performed in three steps: First, we determined the ranks of the four single BRF variables. We started with four ranks per variable. We collapsed the four values each of body overweight and physical inactivity into three based on the number of death cases per group. A score 0 to 3 for each of tobacco smoking and alcohol drinking and a score 0 to 2 for body mass index and for physical inactivity turned out. The four ranks of tobacco smoking were: 0 never or ever less than daily smoker, 1 former daily smoker, 2 current daily smoker less than 20 cigarettes per day, 3 current daily smoker 20 or more cigarettes per day. Alcohol drinking included: 0 AUDIT-C = 1–4, 1 AUDIT-C = 5–7, 2 AUDIT-C alcohol abstinent, 3 AUDIT-C = 8–12. The ranks of body overweight were: 0 BMI < 27, 1 BMI 27- < 32, 2 BMI 32 or higher. Physical inactivity had the ranks: 0 physical inactivity score 16 or lower, 1 physical inactivity score 17–19, 2 physical inactivity score 20.

Second, the BRF sum score was constructed as the sum of the values of the four single variables (0 to 10). The values 7 to 10 included 117 persons only and were collapsed to one group. Thus, the final BRF sum score had eight ranks (0–7). We built the 16 combinations of the four BRFs after dichotomizing tobacco smoking into never daily smokers vs. ever daily smokers, alcohol drinking into low risk (AUDIT-C: 1—4) vs. high risk drinking (AUDIT-C: 5–12) or alcohol abstinence in the last 12 months prior to the interview. We used alcohol abstinence and AUDIT-C 5–12 as one group because evidence had shown that alcohol abstinence included former problem drinkers and had been related to death in a similar way as high alcohol consumption^39^. Body overweight was used as normal weight (BMI lower than 27) vs. overweight (BMI 27 or higher), and physical inactivity as physically active (physical inactivity score: lower than 17) vs. physically inactive (physical inactivity score: 17 or higher).

Third, we analyzed the prediction of all-cause, cardiovascular and cancer mortality by the single BRFs, the BRF sum score, and the BRF combinations. For Cox Proportional Hazards models the proportional hazards assumption was tested using Schoenfeld residuals^40,41^. The Cox Proportional Hazards assumption was not fulfilled in one or more variables in each of the models that have been calculated. Therefore, we used logistic regression analysis. Odds ratios (ORs) with 95% confidence intervals (CIs) are presented. With respect to the single BRFs (Table 3), we analyzed three models. Model 1 included each single BRF adjusted for age and sex. Model 2 was adjusted for age and sex and the three further BRFs. Model 3 in addition was adjusted for mental disorders. The BRF sum score and combinations (Table 4) were analyzed by two models. Model 1 was adjusted for age and sex. Model 2 was adjusted for age, sex, and mental disorders. We used three groups of mental disorders in the data analysis: mood, anxiety, and somatoform disorders. Mood disorders included major depression, dysthymia, bipolar disorders, and mood disorders based on a medical condition. Anxiety disorders were panic disorders, phobias, anxiety disorders not further specified, compulsive disorders, posttraumatic stress disorder, and anxiety disorder on grounds of a medical condition. Somatoform disorders included somatization disorder, conversion disorder, pain disorder, and hypochondriasis.

Missing values were found in the number of cigarettes per day among the current daily smokers (14; 0.35% of 4028) and in the alcohol drinking (35; 0.87% of 4028), body overweight (30; 0.74% of 4028), and physical inactivity variables (40; 0.99% of 4028). The missing values were replaced by the means of 5-year age groups among females and males each. For sex, age and mental disorders no values were missing due to the rules of the computer-assisted interview. They included that the study participant was recontacted if any information in the interview was missing and could not be provided by answers to other questions in the interview^37^. The datasets used and analyzed during the current study are available from the corresponding author on reasonable request. All methods were performed in accordance with the relevant guidelines and regulations. All data were analyzed using Stata 17.0^42^.

### Ethics approval and consent to participate

The ethics committee of the University Medicine Greifswald gave approval for the study (BB 044/13). All study participants had been invited to participate in the study and informed that participation is on their own choice and that they were free to withdraw at any time. All study participants gave informed consent to the scientific use of their data including analysis and publication. They had been informed to withdraw their consent at any time.

## Results

At baseline, 49.90% of the final sample had a BRF sum score 3 or greater (2010 study participants; Table 1). The likelihood of a mental disorder was higher for all BRF sum score ranks compared to no BRF. The data revealed an OR 1.86 (1.32–2.63) for a mental disorder among the persons with two BRFs who were daily smokers and alcohol high risk drinkers. Among the study participants with three BRFs, those who were daily smokers, alcohol high risk drinkers and physically inactive had an OR 2.38 (1.74–3.25) for a mental disorder. The BRFs were associated with each other except tobacco smoking with body overweight (Table 2).

**Table 1:** Characteristics of the sample at baseline: Study participants with mental disorder among all study participants with characteristic.

Characteristic	Total		Participants with mental disorder			
	N	Column %	n	Row %	OR	CI
Males	2022	50.20	413	20.43		
Females	2006	49.80	801	39.93		
Total	4028	100.00				
Tobacco smoking					1.20	1.13–1.27
Never, ever less than daily	1596	39.62	418	26.19	ref	
Former daily	839	20.83	260	30.99	1.56	1.29–1.90
Current daily < 20 cigarettes/day	485	12.04	158	32.58	1.39	1.11–1.74
Current daily > 19 cigarettes/day	1108	27.51	378	34.12	1.81	1.52–2.16
Total	4028	100.00				
Alcohol drinking last 12 months					1.08	0.99–1.18
AUDIT-C 1–4	2877	71.43	878	30.52	ref	
AUDIT-C 5–7	611	15.17	164	26.84	1.21	0.98–1.48
AUDIT-C abstinent	447	11.10	149	33.33	1.16	0.93–1.44
AUDIT-C 8–12	93	2.31	23	24.73	1.15	0.70–1.87
Total	4028	100.00				
Body overweight					1.09	0.96–1.23
Body mass index < 27	3001	74.50	912	30.39	ref	
Body mass index 27- < 32	824	20.46	228	27.67	1.01	0.85–1.21
Body mass index 32 or higher	203	5.04	74	36.45	1.32	0.97–1.79
Total	4028	100.00				
Physical inactivity score					1.18	1.06–1.31
8–16	2032	50.45	603	29.68	ref	
17–19	1574	39.08	486	30.88	1.26	1.09–1.47
20	422	10.48	125	29.62	1.28	1.01–1.63
Total	4028	100.00				
Behavior-related health risk factor sum score					1.14	1.09–1.18
0	594	14.75	153	25.76	ref	
1	718	17.83	203	28.27	1.33	1.03–1.71
2	706	17.53	211	29.89	1.51	1.18–1.94
3	725	18.00	230	31.72	1.66	1.29–2.13
4	615	15.27	198	32.20	1.89	1.46–2.45
5	386	9.58	126	32.64	2.06	1.53–2.76
6	167	4.15	53	31.74	2.06	1.40–3.04
7*	117	2.90	40	34.19	2.73	1.76–4.25
Total	4028	100.00				
Behavior-related health risk factor combinations						
No behavior-related health risk factor	594	14.75	153	25.76	ref	
One behavior-related health risk factor						
1 Daily smoking**	593	14.72	214	36.09	1.73	1.34–2.24
2 Alcohol high risk***	132	3.28	32	24.24	1.05	0.67–1.64
3 Body overweight	138	3.43	39	28.26	1.22	0.80–1.87
4 Physical inactivity	406	10.08	105	25.86	1.25	0.93–1.69
Two behavior-related health risk factors						
5 Daily smoking + alcohol high risk	238	5.91	73	30.67	1.86	1.32–2.63
6 Alcohol high risk + body overweight	48	1.19	10	20.83	0.82	0.39–1.72
7 Body overweight + physical inactivity	113	2.81	37	32.74	1.57	1.01–2.46
8 Daily smoking + physical inactivity	611	15.17	200	32.73	1.83	1.41–2.37
9 Alcohol high risk + physical inactivity	108	2.68	29	26.85	1.47	0.91–2.37
10 Daily smoking + body overweight	182	4.52	58	31.87	1.75	1.20–2.55
Three behavior-related health risk factors						
11 Daily smoking + alcohol high risk + body overweight	107	2.66	24	22.43	1.34	0.81–2.23
12 Alcohol high risk + body overweight + physical inactivity	57	1.42	13	22.81	1.23	0.63–2.39
13 Daily smoking + alcohol high risk + physical inactivity	319	7.92	106	33.23	2.38	1.74–3.25
14 Daily smoking + body overweight + physical inactivity	240	5.96	72	30.00	1.83	1.30–2.59
Four behavior-related health risk factors						
15 Daily smoking + alcohol high risk + body overweight + physical inactivity	142	3.53	49	34.51	2.90	1.92–4.38

Logistic regression analysis for study participants with any of mood, anxiety or somatoform disorder in life before baseline adjusted for age and sex. N number of study participants at baseline, n number of study participants with mental disorder.

*OR* odds ratio, *CI 95*% confidence interval, *ref* reference group, *AUDIT*-C Alcohol use disorders identification test consumption.

*includes the original values 7–10, **ever daily smoking, ***alcohol high risk drinking or alcohol abstinence last 12 months.

**Table 2:** Associations among the behavior-related health risk factors at baseline.

	Tobacco smoking	Alcohol drinking	Body overweight
Alcohol drinking	0.12 p < 0.001	–	–
Body overweight	0.02 ns	0.08 p < 0.001	–
Physical inactivity score	0.15 p < 0.001	0.07 p < 0.001	0.05 p < 0.01

Spearman correlation coefficients.

*p* probability of error, *ns* not significant.

All-cause mortality was predicted by each of the single BRFs after adjustment for age, sex, and the other three BRFs (Table 3). The OR per rank was 1.47 (1.35–1.60) for tobacco smoking and 1.45 (1.30–1.62) for alcohol drinking in model 2. For cardiovascular mortality, the data revealed increased ORs for all four BRFs. The highest rank of alcohol drinking had an OR 4.78 (2.43–9.41) in model 2.

**Table 3:** Single behavior-related health risk factors and mortality.

	N	Deceased		Model 1		Model 2		Model 3	
		n	%	OR	CI	OR	CI	OR	CI
All-cause mortality									
Tobacco smoking				1.50	1.39–1.63	1.47	1.35–1.60	1.47	1.35–1.60
Never, ever less than daily	1596	163	10.21	ref		ref		ref	
Former daily	839	120	14.30	1.03	0.78–1.36	1.01	0.77–1.34	1.02	0.77–1.35
Current daily < 20 cigarettes/day	485	68	14.02	2.01	1.44–2.80	2.04	1.46–2.86	2.03	1.45–2.84
Current daily > 19 cigarettes/day	1108	222	20.04	3.17	2.46–4.07	3.01	2.32–3.90	3.00	2.32–3.89
Alcohol drinking last 12 months				1.52	1.36–1.69	1.45	1.30–1.62	1.45	1.30–1.62
AUDIT-C 1–4	2877	329	11.44	ref		ref		ref	
AUDIT-C 5–7	611	97	15.88	1.33	1.02–1.75	1.18	0.89–1.56	1.18	0.89–1.56
AUDIT-C abstinent	447	119	26.62	2.33	1.80–3.03	2.23	1.71–2.92	2.24	1.72–2.93
AUDIT-C 8–12	93	28	30.11	3.65	2.20–6.07	2.75	1.63–4.65	2.74	1.62–4.62
Body overweight				1.26	1.08–1.47	1.24	1.05–1.45	1.24	1.06–1.45
Body mass index < 27	3001	362	12.06	ref		ref		ref	
Body mass index 27- < 32	824	168	20.39	1.25	1.002–1.55	1.22	0.98–1.54	1.23	0.98–1.54
Body mass index 32 or higher	203	43	21.18	1.62	1.11–2.37	1.66	1.13–2.44	1.67	1.14–2.46
Physical inactivity score				1.28	1.11–1.48	1.13	0.98–1.31	1.13	0.98–1.31
8–16	2032	271	13.34	ref		ref		ref	
17–19	1574	224	14.23	1.18	0.96–1.45	1.04	0.84–1.29	1.04	0.84–1.29
20	422	78	18.48	1.76	1.30–2.39	1.34	0.97–1.84	1.34	0.97–1.84
Cardiovascular mortality									
Tobacco smoking				1.49	1.32–1.69	1.46	1.29–1.66	1.45	1.28–1.65
Never, ever less than daily	1496	63	4.21	ref		ref		ref	
Former daily	783	64	8.17	1.34	0.91–1.98	1.34	0.90–2.01	1.33	0.89–1.99
Current daily < 20 cigarettes/day	440	23	5.23	1.87	1.11–3.15	1.93	1.13–3.30	1.92	1.13–3.28
Current daily > 19 cigarettes/day	970	84	8.66	3.31	2.27–4.82	3.24	2.18–4.82	3.19	2.14–4.75
Alcohol drinking				1.80	1.55–2.09	1.72	1.48–2.01	1.71	1.47–2.00
AUDIT-C 1–4	2666	118	4.43	ref		ref		ref	
AUDIT-C 5–7	554	40	7.22	1.52	1.02–2.27	1.32	0.88–2.00	1.32	0.88–2.00
AUDIT-C abstinent	389	61	15.68	3.23	2.26–4.62	3.15	2.19–4.54	3.13	2.17–4.51
AUDIT-C 8–12	80	15	18.75	6.08	3.14–11.78	4.78	2.43–9.41	4.69	2.37–9.26
Body overweight				1.50	1.21–1.85	1.46	1.17–1.82	1.46	1.17–1.82
Body mass index < 27	2780	141	5.07	ref		ref		ref	
Body mass index 27- < 32	720	64	8.89	1.12	0.81–1.55	1.06	0.76–1.50	1.07	0.76–1.50
Body mass index 32 or higher	189	29	15.34	2.97	1.87–4.73	2.98	1.85–4.81	2.97	1.84–4.80
Physical inactivity score				1.34	1.09–1.65	1.18	0.95–1.46	1.17	0.94–1.45
8–16	1870	109	5.83	ref		ref		ref	
17–19	1443	93	6.44	1.25	0.92–1.70	1.11	0.81–1.53	1.11	0.80–1.52
20	376	32	8.51	1.90	1.22–2.97	1.39	0.87–2.22	1.38	0.86–2.21
Cancer mortality									
Tobacco smoking				1.58	1.40–1.77	1.55	1.38–1.75	1.55	1.37–1.75
Never, ever less than daily	1506	73	4.85	ref		ref		ref	
Former daily	762	43	5.64	0.94	0.63–1.42	0.93	0.61–1.41	0.93	0.61–1.40
Current daily < 20 cigarettes/day	446	29	6.50	2.02	1.27–3.23	2.04	1.27–3.28	2.03	1.27–3.26
Current daily > 19 cigarettes/day	982	96	9.78	3.54	2.49–5.05	3.38	2.35–4.85	3.35	2.33–4.82
Alcohol drinking last 12 months				1.32	1.12–1.54	1.27	1.08–1.49	1.27	1.08–1.49
AUDIT-C 1–4	2702	154	5.70	ref		ref		ref	
AUDIT-C 5–7	551	37	6.72	1.19	0.80–1.77	1.05	0.70–1.58	1.04	0.69–1.57
AUDIT-C abstinent	370	42	11.35	1.78	1.22–2.59	1.70	1.16–2.50	1.70	1.16–2.50
AUDIT-C 8–12	73	8	10.96	2.29	1.03–5.09	1.59	0.70–3.64	1.58	0.69–3.61
Body overweight				1.20	0.96–1.51	1.17	0.93–1.47	1.17	0.93–1.48
Body mass index < 27	2787	148	5.31	ref		ref		ref	
Body mass index 27- < 32	741	85	11.47	1.62	1.21–2.18	1.60	1.18–2.17	1.61	1.19–2.18
Body mass index 32 or higher	168	8	4.76	0.72	0.34–1.52	0.77	0.36–1.62	0.76	0.36–1.62
Physical inactivity score				1.26	1.03–1.54	1.12	0.91–1.38	1.12	0.91–1.38
8–16	1883	122	6.48	ref		ref		ref	
17–19	1437	87	6.05	1.10	0.81–1.48	0.97	0.71–1.32	0.97	0.71–1.31
20	376	32	8.51	1.78	1.15–2.74	1.40	0.89–2.19	1.39	0.89–2.18

N number of study participants at baseline. n number of study participants who had been deceased in the time after baseline until mortality follow-up. Model 1: adjusted for age and sex. Model 2: adjusted for age and sex, and the other three behavior-related health risk factors at baseline. Model 3: adjusted for age, sex, the other three behavior-related health risk factors, and three groups of mental disorder (mood disorder, anxiety disorder, somatoform disorder) in life before baseline. Logistic regression due to Cox Proportional Hazards Assumption not having been fulfilled in one or more single variables.

*CI* 95% confidence interval, *OR* odds ratio, *ref* reference group, *AUDIT*-*C* Alcohol use disorders identification test consumption.

The higher the BRF sum score was, the higher were the proportions of deceased persons (Table 4). Among the study participants who had no BRF, 7.91%, among the study participants who had seven to ten BRFs, 34.19% had been deceased. The data revealed 38% higher odds of all-cause mortality for each of the ranks, the study participants who had none of the BRFs being the reference. For persons with a sum score 3 or higher, the ORs were significantly increased compared to the persons who had no BRF. After adjustment for mental disorders in addition to age and sex, the OR per sum score rank was 1.38 (1.31–1.46) and 8.09 (4.66–14.02) for the sum score rank 7 to 10. Mood, anxiety and somatoform disorders were not related with an increased mortality risk.

**Table 4:** Behavior-related health risk factor sum score, health risk factor combinations and mortality.

	N	Deceased		Model 1		Model 2	
		n	%	OR	CI	OR	CI
All-cause mortality							
Behavior-related health risk factor sum score				1.38	1.31–1.46	1.38	1.31–1.46
0	594	47	7.91	ref		ref	
1	718	58	8.08	1.02	0.67–1.56	1.02	0.67–1.55
2	706	85	12.04	1.42	0.96–2.11	1.42	0.95–2.10
3	725	100	13.79	2.06	1.39–3.04	2.05	1.39–3.04
4	615	109	17.72	2.94	1.98–4.37	2.93	1.97–4.35
5	386	90	23.32	4.23	2.78–6.41	4.23	2.78–6.44
6	167	44	26.35	5.55	3.35–9.18	5.54	3.34–9.18
7*	117	40	34.19	8.10	4.69–14.00	8.09	4.66–14.02
Mood disorder				–		1.17	0.86–1.60
Anxiety disorder				–		0.90	0.67–1.21
Somatoform disorder				–		0.98	0.73–1.31
Behavior-related health risk factor combinations							
No behavior-related risk factor	594	47	7.91	ref		ref	
One behavior-related risk factor							
1 Daily smoking**	593	69	11.64	1.85	1.23–2.80	1.84	1.22–2.78
2 Alcohol high risk***	132	16	12.12	1.22	0.65–2.31	1.20	0.64–2.27
3 Body overweight	138	17	12.32	1.29	0.69–2.38	1.28	0.69–2.38
4 Physical inactivity	406	23	5.67	0.84	0.49–1.45	0.84	0.49–1.43
Two behavior-related health risk factors							
5 Daily smoking + alcohol high risk	238	44	18.49	2.94	1.82–4.76	2.91	1.80–4.70
6 Alcohol high risk + body overweight	48	11	22.92	2.48	1.15–5.35	2.48	1.15–5.36
7 Body overweight + physical inactivity	113	17	15.04	1.89	1.01–3.53	1.89	1.01–3.53
8 Daily smoking + physical inactivity	611	71	11.62	1.92	1.26–2.91	1.90	1.25–2.88
9 Alcohol high risk + physical inactivity	108	16	14.81	2.30	1.19–4.44	2.29	1.18–4.41
10 Daily smoking + body overweight	182	39	21.43	2.39	1.46–3.93	2.38	1.45–3.91
Three behavior-related health risk factors							
11 Daily smoking + alcohol high risk + body overweight	107	28	26.17	2.76	1.56–4.87	2.77	1.57–4.90
12 Alcohol high risk + body overweight + physical inactivity	57	16	28.07	4.20	2.07–8.53	4.24	2.09–8.62
13 Daily smoking + alcohol high risk + physical inactivity	319	76	23.82	4.82	3.11–7.48	4.79	3.08–7.43
14 Daily smoking + body overweight + physical inactivity	240	46	19.17	2.45	1.53–3.93	2.44	1.52–3.91
Four behavior-related health risk factors							
15 Daily smoking + alcohol high risk + body overweight + physical inactivity	142	37	26.06	3.41	2.01–5.76	3.38	1.99–5.73
Cardiovascular mortality							
Behavior-related health risk factor sum score				1.49	1.37–1.62	1.48	1.37–1.61
0	564	17	3.01	ref		ref	
1	680	20	2.94	0.95	0.48–1.88	0.94	0.48–1.86
2	654	33	5.05	1.47	0.79–2.74	1.45	0.78–2.70
3	662	37	5.59	2.14	1.15–3.95	2.10	1.13–3.88
4	554	48	8.66	3.56	1.94–6.54	3.51	1.91–6.45
5	335	39	11.64	5.29	2.81–9.97	5.17	2.74–9.76
6	146	23	15.75	8.79	4.29–18.01	8.54	4.16–17.56
7*	94	17	18.09	11.70	5.22–26.22	11.29	5.01–25.44
Mood disorder				–		1.12	0.70–1.80
Anxiety disorder				–		1.00	0.65–1.55
Somatoform disorder				–		1.22	0.80–1.85
Behavior-related health risk factor combinations							
No behavior-related health risk factor	564	17	3.01	ref		ref	
One behavior-related health risk factor							
1 Daily smoking**	549	25	4.55	1.87	0.97–3.59	1.82	0.95–3.50
2 Alcohol high risk***	120	4	3.33	0.81	0.26–2.51	0.80	0.26–2.50
3 Body overweight	128	7	5.47	1.36	0.53–3.44	1.37	0.54–3.47
4 Physical inactivity	389	6	1.54	0.64	0.25–1.67	0.63	0.24–1.66
Two behavior-related health risk factors							
5 Daily smoking + alcohol high risk	214	20	9.35	3.94	1.93–8.05	3.82	1.87–7.81
6 Alcohol high risk + body overweight	43	6	13.95	3.77	1.35–10.54	3.72	1.33–10.39
7 Body overweight + physical inactivity	104	8	7.69	2.57	1.05–6.31	2.57	1.05–6.31
8 Daily smoking + physical inactivity	563	23	4.09	1.78	0.91–3.47	1.72	0.88–3.38
9 Alcohol high risk + physical inactivity	99	7	7.07	2.71	1.04–7.05	2.58	0.99–6.74
10 Daily smoking + body overweight	156	13	8.33	1.99	0.91–4.36	1.92	0.88–4.22
Three behavior-related health risk factors							
11 Daily smoking + alcohol high risk + body overweight	96	17	17.71	4.40	2.04–9.49	4.32	2.00–9.32
12 Alcohol high risk + body overweight + physical inactivity	49	8	16.33	6.02	2.29–15.82	5.80	2.19–15.33
13 Daily smoking + alcohol high risk + physical inactivity	282	39	13.83	7.13	3.73–13.65	6.89	3.59–13.21
14 Daily smoking + body overweight + physical inactivity	213	19	8.92	2.82	1.38–5.77	2.75	1.34–5.63
Four behavior-related health risk factors							
15 Daily smoking + alcohol high risk + body overweight + physical inactivity	120	15	12.50	3.81	1.74–8.33	3.69	1.68–8.10
Cancer mortality							
Behavior-related health risk factor sum score				1.35	1.25–1.46	1.35	1.25–1.46
0	570	23	4.04	ref		ref	
1	687	27	3.93	1.04	0.58–1.87	1.04	0.58–1.86
2	662	41	6.19	1.55	0.90–2.67	1.53	0.89–2.64
3	669	44	6.58	2.11	1.23–3.63	2.10	1.22–3.61
4	546	40	7.33	2.55	1.46–4.47	2.51	1.43–4.41
5	331	35	10.57	4.02	2.24–7.20	3.98	2.21–7.14
6	138	15	10.87	4.70	2.27–9.75	4.69	2.26–9.73
7*	93	16	17.20	8.58	4.03–18.27	8.38	3.93–17.89
Mood disorder				–		1.17	0.76–1.80
Anxiety disorder				–		1.10	0.74–1.65
Somatoform disorder				–		0.86	0.56–1.32
Behavior-related health risk factor combinations							
No behavior-related health risk factor	570	23	4.04	ref		ref	
One behavior-related health risk factor							
1 Daily smoking**	557	33	5.92	1.93	1.10–3.39	1.91	1.08–3.36
2 Alcohol high risk***	124	8	6.45	1.33	0.57–3.14	1.31	0.56–3.09
3 Body overweight	129	8	6.20	1.23	0.53–2.89	1.24	0.53–2.89
4 Physical inactivity	394	11	2.79	0.90	0.43–1.90	0.89	0.42–1.88
Two behavior-related health risk factors							
5 Daily smoking + alcohol high risk	210	16	7.62	2.53	1.26–5.06	2.48	1.23–4.98
6 Alcohol high risk + body overweight	42	5	11.90	2.38	0.83–6.81	2.30	0.80–6.62
7 Body overweight + physical inactivity	103	7	6.80	1.72	0.70–4.22	1.71	0.69–4.21
8 Daily smoking + physical inactivity	571	31	5.43	1.97	1.10–3.51	1.93	1.08–3.46
9 Alcohol high risk + physical inactivity	97	5	5.15	1.66	0.59–4.66	1.66	0.59–4.66
10 Daily smoking + body overweight	164	21	12.80	2.86	1.49–5.48	2.84	1.48–5.45
Three behavior-related health risk factors							
11 Daily smoking + alcohol high risk + body overweight	87	8	9.20	2.05	0.85–4.93	2.05	0.85–4.94
12 Alcohol high risk + body overweight + physical inactivity	47	6	12.77	3.18	1.16–8.66	3.23	1.18–8.81
13 Daily smoking + alcohol high risk + physical inactivity	264	21	7.95	3.36	1.74–6.49	3.32	1.72–6.42
14 Daily smoking + body overweight + physical inactivity	214	20	9.35	2.64	1.37–5.07	2.61	1.36–5.04
Four behavior-related health risk factors							
15 Daily smoking + alcohol high risk + body overweight + physical inactivity	123	18	14.63	4.14	2.04–8.40	4.06	2.00–8.26

N number of study participants at baseline. n number of study participants who had been deceased in the time after baseline until mortality follow-up. Model 1: adjusted for age and sex. Model 2: adjusted for age and sex and three groups of mental disorder (mood disorder, anxiety disorder, somatoform disorder) in life before baseline. Logistic regression due to Cox Proportional Hazards Assumption not having been fulfilled in one or more single variables. Behavior-related health risk factor sum score: sum of ranks of tobacco smoking, alcohol drinking, body overweight, and physical inactivity.

*OR* odds ratio, CI 95%-confidence interval, ref reference group.

– not applicable. *includes the original values 7–10, **ever daily smoking, ***alcohol high risk drinking or alcohol abstinence last 12 months.

For the combinations of the single BRFs, the study participants who had no BRFs were the reference group. Among the person groups with two or more BRFs, all ORs for all-cause mortality were significantly increased. Among the participants with two BRFs, ever daily smokers and alcohol high risk drinkers had the highest OR (2.91; 1.80–4.70) after adjustment for age, sex, and mental disorders. The lowest OR was found in the group that had neither a tobacco- nor an alcohol-related BRF. Among the persons with three BRFs, those with ever daily smoking, alcohol high risk drinking and physical inactivity had the highest OR (4.79; 3.08–7.43). All ORs that were statistically significant after adjustment for age and sex remained to be significant after additional adjustment for mental disorders (model 2).

According to cardiovascular mortality, in both models increased ORs of the BRF sum score were found compared to persons without BRFs. The data revealed 49% higher odds of mortality for each of the ranks with the study participants who had none of the BRFs as the reference (model 1). The data of the combinations revealed that among persons who had three or four BRFs all combinations showed significantly increased ORs.

According to cancer mortality, in model 1 increased ORs were found for the BRF sum score (1.35; 1.25–1.46). Persons with any mood, anxiety or somatoform disorder did not have an increased risk of cancer death. For the combinations of the BRFs, the data revealed eight increased ORs. Among these, seven included ever daily smoking. Ever daily smokers turned out to be the only subgroup with an increased OR among the study participants with one BRF. The highest OR (4.14; 2.04–8.40) was found for those who had four BRFs.

## Discussion

This study has four main findings. First, the BRF sum score turned out to be related with mortality risk in a dose-dependent manner. Second, among the specific combinations of the single BRFs alcohol drinking and tobacco smoking seem to strongly contribute to the prediction of mortality. Third, cardiovascular mortality was predicted by the BRFs in a particularly strong manner. Fourth, mental disorders did not change the findings considerably.

The BRF sum score predicted mortality. The score turned out to have three advantages. First, it gave evidence for the dose-dependent relation with mortality. Each of the seven ranks indicated a 38% higher likelihood to die within the 20 years. This finding gives evidence on a dose-relation between BRFs and mortality risk. Many of the studies before had been limited to BRFs as being present or absent^8,9,43^. Second, the sum score may provide more information about risk of death than single BRFs because of covering four behavior-related health risk factors and eight ranks. Third, the data revealed rank 3 of the sum score as the lowest one that indicates an increased risk of death. This finding seems to be particularly important for public health. Among all 4028 study participants, 49.90% had a BRF sum score 3 or higher.

According to specific combinations, the findings suggest that alcohol consumption might be of particularly strong influence. Persons with high risk alcohol drinking had seven ORs greater than 2 for all-cause mortality. This was the largest number of ORs greater than 2 among all BRFs. High risk alcohol drinking was also involved in all three combinations of the BRFs with an OR larger than 3.

According to cardiovascular mortality, the BRF sum score turned out to be related with the risk of death in a particularly high dose manner. Each rank of the sum score was 48% higher compared to the respective lower rank after adjustment for age, sex and mental disorders. Nine combinations of BRFs had an OR larger than 2. Among them, the most frequent BRF was alcohol high risk drinking. Persons with high risk drinking were involved in seven of the nine ORs larger than 2. According to cancer mortality, the BRF sum score was also in a linear relation to the mortality risk with a 35% higher risk per rank after adjustment for age and sex. Daily tobacco smoking was included in seven out of eight BRF combinations that predicted cancer mortality. Tobacco smoking was the only BRF that predicted mortality among persons with one BRF. This lends support to the assumption that in cancer mortality tobacco smoking might have a particularly strong influence among the four BRFs.

Mood, anxiety or somatoform disorders did not add to the prediction of mortality by BRFs. Our findings at first view seem to contradict findings of a higher mortality among subjects with a mental disorder than among healthy comparison samples^16^. One reason might be that our sample had been drawn from the general population. The study participants with a mental disorder in their majority included those who had not utilized psychiatric treatment. Among them, there might be those with a low severity of the disorder. Persons with a mental disorder but no treatment survived longer than those with a treated mental disorder^34^.

Strengths of our study include that a sum score with the potential of ten ranks has been used. This provides the opportunity to detect more detailed dose-relations between BRFs and mortality. Second, 15 combinations of the BRFs in addition to no BRF turned out to add information to the relations between BRFs and mortality. Subgroups of persons at particularly high risk among the general population and combinations of single BRFs that might be more important than others in the prediction of mortality have been detected. Third, we assessed mental disorders using an internationally standardized interview and the diagnostic criteria of the American Psychiatric Association. Our study added findings about BRFs, mental disorders, all-cause, cardiovascular, and cancer mortality in the time frame of 20 years. Limitations of our study include that we had self-report data only. Reporting bias is likely. This particularly might be the case for alcohol consumption. But for the other BRFs also, underreporting may have taken place. It seems plausible that residents with high ranks of BRFs might be particularly prone to underreport BRFs. Feelings of guilt and shame might be responsible for that. Our study was limited to four BRFs. More may be relevant for death. BRFs may have evolved or been discontinued during the 20 years. We reported a considerable number of statistical test results. Problems of multiple testing should be kept in mind.

## Conclusions

The findings of this 20-year mortality follow-up study suggest that the eight ranks of the sum score of behavior-related health risk factors are related to risk of death in a dose-dependent manner. This was found for all-cause, cardiovascular and cancer mortality. The study results provide evidence that especially alcohol high risk drinking and tobacco smoking might have a robust influence on mortality. Cardiovascular mortality was predicted by the BRFs in a particularly strong manner. Lifetime mood, anxiety or somatoform disorders might not have an effect on mortality in addition to the behavior-related health risk factors.

## Data Availability

The datasets used and analyzed during the current study are available from the corresponding author on reasonable request.
